# Extraction and Inhibition of Enzymatic Activity of Botulinum Neurotoxins/A1, /A2, and /A3 by a Panel of Monoclonal Anti-BoNT/A Antibodies

**DOI:** 10.1371/journal.pone.0005355

**Published:** 2009-04-28

**Authors:** Suzanne R. Kalb, Jianlong Lou, Consuelo Garcia-Rodriguez, Isin N. Geren, Theresa J. Smith, Hercules Moura, James D. Marks, Leonard A. Smith, James L. Pirkle, John R. Barr

**Affiliations:** 1 Centers for Disease Control and Prevention, National Center for Environmental Health, Division of Laboratory Sciences, Atlanta, Georgia, United States of America; 2 Department of Anesthesia and Pharmaceutical Chemistry, University of California at San Francisco, San Francisco General Hospital, San Francisco, California, United States of America; 3 Integrated Toxicology, United States Army Medical Research Institute of Infectious Diseases (USAMRIID), Ft. Detrick, Maryland, United States of America; Baylor College of Medicine, United States of America

## Abstract

Botulinum neurotoxins (BoNTs) are extremely potent toxins that are capable of causing death or respiratory failure leading to long-term intensive care. Treatment includes serotype-specific antitoxins, which must be administered early in the course of the intoxication. Rapidly determining human exposure to BoNT is an important public health goal. In previous work, our laboratory focused on developing Endopep-MS, a mass spectrometry-based endopeptidase method for detecting and differentiating BoNT/A–G serotypes in buffer and BoNT/A, /B, /E, and /F in clinical samples. We have previously reported the effectiveness of antibody-capture to purify and concentrate BoNTs from complex matrices, such as clinical samples. Because some antibodies inhibit or neutralize the activity of BoNT, the choice of antibody with which to extract the toxin is critical. In this work, we evaluated a panel of 16 anti-BoNT/A monoclonal antibodies (mAbs) for their ability to inhibit the *in vitro* activity of BoNT/A1, /A2, and /A3 complex as well as the recombinant LC of A1. We also evaluated the same antibody panel for the ability to extract BoNT/A1, /A2, and /A3. Among the mAbs, there were significant differences in extraction efficiency, ability to extract BoNT/A subtypes, and inhibitory effect on BoNT catalytic activity. The mAbs binding the C-terminal portion of the BoNT/A heavy chain had optimal properties for use in the Endopep-MS assay.

## Introduction

Botulinum neurotoxins (BoNTs) are protein toxins produced by some species of the genus *Clostridium*, in particular, *Clostridium botulinum, C. butyricum*, *C. baratii, and C. argentinense.* Intoxication with one of the seven distinct serotypes of BoNT (A–G) causes botulism, a disease that is contracted by ingestion of food containing the toxin [1], [2], colonization of the bacteria in the gastrointestinal tract of infants or immunocompromised individuals, inhalation of the toxin, or contact of the bacterium with a wound [1]. Due to its high toxicity, availability, and ease of preparation, it is considered a likely agent for bioterrorism [3]. Treatment of botulism involves administration of therapeutic immunoglobulin product and is most effective when administered within 24 hr of exposure [1]. However, the currently licensed antitoxins are serotype-specific, mainly for BoNT/A and/or BoNT/B, while investigational BoNT/E and BoNT/A–G are also available. Since these products will not protect the patient if the botulism is caused by any of the other serotypes, rapidly determining exposure to BoNT and serotyping the toxin involved are critical to choose the right antitoxin for treating the patient.

BoNTs are zinc metalloproteases which cleave and therefore inactivate proteins which are necessary for acetylcholine release. Each serotype of BoNT consists of a heavy chain (HC) of approximately 100,000 daltons and a light chain (LC) of about 50,000 daltons. The heavy chain is responsible for both receptor binding via its C-terminal (CT) binding domain [4], [5] (H_C_) and delivering the catalytic light chain (LC) to its target via its N-terminal translocation domain (H_N_) [6]. The LC selectively cleaves neuronal proteins required for acetylcholine release. Although the LC accounts for the specific toxicity, it requires the heavy chain to produce this toxic activity *in vivo*. BoNT/A, /C, and /E cleave SNAP (synaptosomal-associated protein)-25 while BoNT/B, /D, /F, /G and the closely related tetanus toxin all cleave synaptobrevin 2 (also called VAMP 2). Of the serotypes, only one, BoNT/C, cleaves more than one specific protein. In addition to cleaving SNAP-25, BoNT/C also cleaves syntaxin [2]. BoNTs are released into the environment by clostridial species in a protein complex consisting of the pure neurotoxin and a number of neurotoxinassociated proteins (BoNT complex).

Previously, our laboratory reported on the development of an assay for BoNT detection and serotype differentiation termed the Endopep-MS method [7]–[12]. This method detects all 7 BoNT serotypes and involves incubating BoNT with a peptide substrate that mimics BoNT's natural, *in vivo* target. Each BoNT cleaves its peptide substrate in a specific location, and that location is different for each of the BoNT serotypes [2], [7], [9], [12]. The reaction mixture is then introduced into a mass spectrometer, which detects and accurately reports the mass of any peptides within the mixture. Detecting the peptide cleavage products corresponding to their specific toxin-dependent location indicates the presence of a particular BoNT serotype, A–G. If the peptide substrate either remains intact, or is cleaved in a location other than the toxin-specific site, then that BoNT serotype is not present at detectable levels. Historically, mouse assays have been the most commonly used method to detect BoNT [13], but as previous publications [7]–[9], [12] have demonstrated, the Endopep-MS method can detect BoNT at levels comparable with or lower than levels detected with mouse bioassays.

As previously reported, Endopep-MS is effective in identifying BoNT/A, /B, /E, and /F in clinical samples. It uses an antibody affinity concentration/purification step prior to reaction with the substrate [9]–[12]. Polyclonal antibodies to BoNT/A, /B, /E, and /F are available commercially and were found to be successful for concentrating and purifying BoNT from a complex matrix. However, because polyclonal antibodies consist of a heterogeneous mixture of antibodies, they may recognize various portions of the BoNT antigen molecule, each with different affinities. By contrast, monoclonal antibodies (mAbs) recognize specific protein epitopes, ensuring that they recognize a single antigenic site, and always with the same affinity. Monoclonal antibodies have recently been produced to BoNT/A [14]–[22] and we explored the use of these high-affinity mAbs to improve the sample preparation portion of the assay.

We also reported that binding polyclonal anti-BoNT/A could interfere with the activity of the LC of BoNT/A as measured by Endopep-MS [9] specifically, because Endopep-MS detects the presence of BoNT by measuring the activity of the light chain. Unfortunately, this can raise the BoNT-detection limit, depending on where the antibodies bind to the toxin. We proposed, therefore, the possibility that the assay might be improved by using selected mAbs that do not bind the LC and thus, do not inhibit the catalytic activity.

Another feature of BoNT/A is that it exhibits genetic and amino acid variance within the toxin type, or serotype. As currently defined, BoNT/A consists of /A1, /A2, /A3, and /A4 subtypes [23]. This variability among the BoNT/A subtypes consists of 15% or less amino acid variance [23] and this variance has been reported to affect binding of the toxin to anti-BoNT/A mAbs [24]. For these reasons, it is important to be able to detect all toxin subtypes because an outbreak of botulism may be attributed to more than just the familiar BoNT/A1 subtype.

Our laboratory has already determined that the Endopep-MS assay can be used to detect all currently known subtypes of BoNT/A [12]. However, the sensitivity of the detection varies with subtype. Our goal in this work is to evaluate a panel of mAbs for their inhibitory and extraction abilities, thus optimizing assay sensitivity with all BoNT/A subtypes. Unfortunately, BoNT/A4 only exists in conjunction with BoNT/bivalent B, and the /A4 component is considerably smaller than the B component. The low concentration of BoNT/A4 makes it difficult to perform multiple experiments, and therefore evaluating mAb extraction and inhibition of BoNT/A4 will not be addressed in this work. Here, we evaluate a panel of 16 monoclonal anti-BoNT/A mAbs for their ability to inhibit the *in vitro* activity of the complex form of BoNT/A1, /A2, and /A3 as well as the recombinantly produced BoNT/A1 LC. We also evaluate the same antibody panel for the ability to extract the complex form of BoNT/A1, /A2, and /A3. The results indicate which mAbs have the optimal properties for use in the Endopep-MS detection of BoNT/A.

## Materials and Methods

### Materials

Botulinum neurotoxin is very toxic and must be handled using care and appropriate safety measures. All neurotoxins were handled in a level 2 biosafety cabinet equipped with HEPA filters. BoNT/A3, strain Loch Maree, crude culture supernatants were produced from *Clostridium botulinum* after growth for 5 days at 35°C. After centrifugation of the culture, supernatant was removed and filtered through 0.22-µm filters. The supernatant was titered using a mouse intraperitoneal (i.p.) endpoint assay to determine specific activity. The assay involves duplicate two-fold dilutions ranging from 20 to 0.156 mouse LD_50_ (mLD_50_), based on initial values of 5×10^4^ to 1×10^5^ mLD_50_. In addition, commercially purified BoNT/A1 (strain Hall) and BoNT/A2 (strain FRI-honey) complex toxins from Metabiologics (Madison, WI) were used for comparative testing.

As described elsewhere [25], recombinant BoNT/A1 LC was expressed and purified. Polyclonal anti-BoNT/A1 rabbit-specific IgGs were provided by Metabiologics (Madison, WI) in 150 mM potassium phosphate (pH 7.4). Monoclonal antibodies 3D12, ING1, and ING2 were generated using display technologies from human volunteers immunized with pentavalent BoNT toxoid containing the BoNT/A1 subtype [17], [22], [26]. C25 was generated from a mouse immunized with BoNT/A1 H_C_ [17], [21]. HuC25 is a humanized version of C25, and AR1, AR2, AR4, CR1, and CR2 are mutants of HuC25 engineered to have higher affinity for BoNT/A1 or better cross reactivity to the BoNT/A2 and /A3 subtypes [15], [19]. B4 and 2A9 were generated from mice transgenic for the human immunoglobulin locus that were immunized with BoNT/A1 H_C_. RAZ1 is a mutant of 3D12 engineered to bind BoNT/A with higher affinity [19]. 2G11 is a mutant of ING1 engineered to bind BoNT/A with higher affinity [26]. 4A1 and 5A20 were generated using display technologies from human volunteers immunized with pentavalent BoNT toxoid containing the BoNT/A1 subtype [26]. 4A1.1 and 5A20.4 are mutants of the human antibodies 4A1 and 5A20 engineered to bind BoNT/A with higher affinity [26]. All of the above mAbs were produced with human IgG1/kappa constant regions recombinantly from Chinese Hamster Ovary cells and purified to greater than 90% homogeneity by protein A affinity chromatography. Purified IgG were buffer exchanged into phosphate buffered saline (PBS) at a concentration of 1 mg/ml, as previously described [16].

Dynabeads® Protein G were purchased from Invitrogen (Carlsbad, CA) at 1.3 g/cm^3^ in phosphate buffered saline, pH 7.4, containing 0.1% Tween®-20 and 0.02% sodium azide. Except where indicated, all chemicals were from Sigma-Aldrich (St. Louis, MO). Peptides were synthesized by Los Alamos National Laboratory (Los Alamos, NM,) and are identical to those reported previously [7]–[12]. Specifically, the peptide substrate has the sequence biotin-KGSNRTRIDQGNQRATRXLGGK-biotin and the internal standard peptide has the sequence R**A**TRXLGGK-biotin where **A** indicates a +7 mass increase to a naturally-occurring alanine.

### BoNT/A Inhibition Experiments

A 2-µL solution containing 30 ng of each titered IgG was added to a 2-µL solution containing 100 mLD_50_ of BoNT/A1, /A2, /A3 or 230 pg recombinant LC BoNT/A1. The mixtures were incubated for 1 hr at room temperature with no agitation. Then, 16 µL of a reaction mixture (0.05 M Hepes [pH 7.3], 25 mM dithiothreitol, 20 µM ZnCl_2_, 1 mg/mL bovine serum albumin, and 50 pmol/µL of peptide substrate) was added to the prior mixture. All samples then were incubated at 37°C for 4 h with no agitation. All assays were performed in triplicate and results were averaged.

### BoNT A Extraction Experiments

The IgG was immobilized and crosslinked to the Dynabeads® Protein G as described in previous publications [9]–[12]. An aliquot of 20 µL of antibody-coated beads was mixed for 1 h with a 0.5-mL solution containing 100 mLD_50_ of either BoNT/A1, /A2, or /A3. The solution was prepared by spiking 2500 mLD_50_ of either BoNT/A1, /A2, or /A3 into 12.5 mL of phosphate buffered saline with 0.01% Tween (PBST) buffer. After mixing for 1 h with constant agitation at room temperature, the beads were washed twice in 1 mL each of PBST and then washed once in 100 µL of water. The beads were reconstituted in a 20-µL solution containing 0.05 M Hepes (pH 7.3), 25 mM dithiothreitol, 20 µM ZnCl_2_, 1 mg/mL bovine serum albumin, and 50 pmol/µL of peptide substrate. All samples then were incubated at 37°C for 4 h with no agitation. All assays were performed in triplicate and results were averaged.

### MS Detection

A master mix was created consisting of 9 parts matrix solution (alpha-cyano-4-hydroxy cinnamic acid) at 5 mg/mL in 50% acetonitrile, 0.1% trifluoroacetic acid (TFA), and 10 mM ammonium phosphate) and 1 part internal standard peptide in water at 5 µM. To 18 µL of this master mix, 2 µL of each reaction supernatant were added. We pipeted 0.5 µL of this mixture onto each spot of a 192-spot matrix-assisted laser desorption/ionization (MALDI) plate (Applied Biosystems, Framingham, MA). Mass spectra of each spot were obtained by scanning from 1100 to 3200 *m/z* in MS-positive ion reflector mode on an Applied Biosystems 4800 Proteomics Analyzer (Framingham, MA). The instrument uses a nitrogen laser at 337 nm, and each spectrum is an average of 2400 laser shots.

## Results

### Inhibition of BoNT/A Activity with Antibody Addition

The mAbs evaluated for their ability to inhibit substrate cleavage by BoNT/A bind 6 different non-overlapping epitopes on the BoNT/A H_C_, H_N_, LC-H_N_ or LC with high affinity (Table 1). Polyclonal antibodies were prepared using the entire BoNT/A devoid of associated nontoxic proteins as immunogen and thus likely contained antibodies binding to all BoNT/A domains.

**Table 1:** A list of mAbs to BoNT/A with their epitopes and affinities for BoNT/A1, A2, and A3 as measured by dissociation rates (K_D_s).

Antibody	BoNT/A epitope	IgG Affinity for BoNT by K_D_ (×10^−12^M^−1^)		
		A1	A2	A3
C25	H_CN_-epitope 1	95.0	NM	NM
HuC25	H_CN_-epitope 1	45.1	19,300	NM
AR1	H_CN_-epitope 1	12.4	>20,000	NM
AR2	H_CN_-epitope 1	6.8	20,100	NM
AR4	H_CN_-epitope 1	1.33	>20,000	NM
CR1	H_CN_-epitope 1	2.48	1730	NM
CR2	H_CN_-epitope 1	10.0	290	150
3D12	H_CC_ epitope 2	60.7	152	NM
RAZ1	H_CC_ epitope 2	1.48	3.69	4.65
2A9	H_CC_ epitope 2	76.4	236.5	NM
B4	H_CC_ epitope 3	95.9	NB	NB
4A1.1	H_N_ epitope 4	11.34	>1000	NM
ING1	L_C_-H_N_ epitope 5	314.3	719.1	400
2G11	L_C_-H_N_ epitope 5	25.1	40.4	18.0
ING2	L_C_ epitope 5	9.57	7.42	NB
5A20.4	L_C_ epitope 6	13.6	NB	NB

NM indicates not measured and NB indicates that it does not bind.

Inhibiting the enzymatic activity of BoNT/A upon addition of antibodies depends upon the antigenic epitopes which the antibody recognizes. Because BoNTs consist of two chains, with one chain responsible for enzymatic activity (LC) and another chain (HC) responsible for directing the enzymatically active light chain to its target, antibodies reacting with the light chain of the toxin may inhibit the toxin's activity. Therefore, an inhibition experiment was performed in which an equal amount of each antibody was added to the BoNT/A Endopep-MS reaction. The molar concentration of antibody in all cases exceeds the molar concentration of toxin by at least 40 to 70 fold and exceeds the dissociation equilibrium constant (K_D_) by at least 300 fold, ensuring that essentially all of the LC is bound by antibody and that antibody activity is evaluated separately from antibody affinity. In this reaction, BoNT/A cleaves the peptide substrate in a specific location, resulting in peptide cleavage products at 1197.7 and 1699.9 *m/z*. Greater levels of toxin or more active toxin will produce more of these peptide cleavage products.

**Figure 1:**
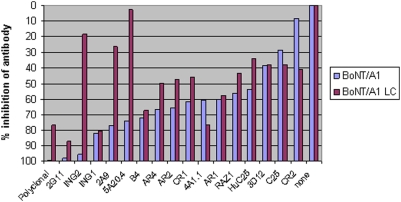
A graph indicating the % of inhibition in activity of BoNT/A1 or /A1 light chain following neutralization with the antibody panel. The sample with no antibodies had no inhibition of activity, so the % of inhibition in activity is calculated by dividing the peak area ratio of the peptide cleavage product over the internal standard peptide of the individual antibody by the peak area ratio of the sample with no antibodies.

Because this experiment involves the use of equal levels of toxin in all cases, the amount of cleavage product is therefore related to the toxin's activity rather than the amount of toxin. Because the internal standard (ISTD) contains the same sequence as the C-terminal (CT) cleavage product and the same amount of ISTD is added to each sample, the ISTD can be used to measure the amount of cleavage product and hence active toxin present in a sample, compared with other samples. This inhibition experiment was performed with a panel of 16 mAbs and one polyclonal antibody preparation against BoNT/A1, /A2, and /A3 toxin complexes and with recombinant LC of BoNT/A1. Table 2 lists the peak area ratios of the native cleavage product over the ISTD obtained from the reaction of these 4 toxins with the panel of 17 antibodies. An increase in peak area ratio indicates a more active toxin. Figure 1 is a graph of percent inhibition of antibody as calculated through the peak area ratios of each antibody compared to the peak area ratio in the absence of antibodies.

**Table 2:** Peak area ratios of the peptide cleavage product over the internal standard peptide obtained from the Endopep-MS reaction of BoNT/A with its peptide substrate in the presence of the antibody panel.

Antibody	BoNT A1	BoNT A1 Light Chain	BoNT A2	BoNT A3
Polyclonal	0.02	0.29	0.01	0.82
2G11	0.12	0.16	0.15	0.98
ING2	0.28	1.02	0.05	3.31
ING1	1.12	0.24	1.79	2.96
2A9	1.25	0.92	1.18	1.94
5A20.4	1.51	1.22	1.43	1.99
B4	1.98	0.41	1.09	2.17
AR4	2.07	0.63	1.51	2.13
AR2	2.12	0.66	1.52	2.28
CR1	2.36	0.68	1.23	2.73
4A1.1	2.42	0.39	1.28	2.64
AR1	2.46	0.53	1.68	3.02
RAZ1	2.71	0.71	0.97	2.49
HuC25	2.85	0.83	1.84	3.14
3D12	3.83	0.78	1.78	3.36
C25	4.45	0.78	2.54	3.51
CR2	5.67	0.74	1.64	3.92
none	6.19	1.25	2.75	4.48

Four different forms of BoNT A were used, and they include /A1, A2, A3, and A1 light chain.

**Figure 2:**
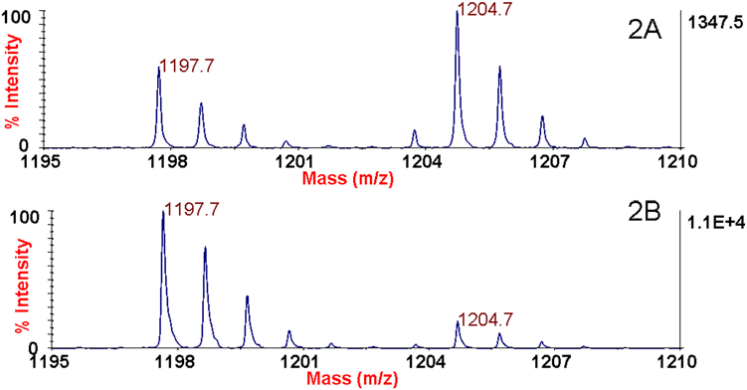
Mass spectra of the Endopep-MS BoNT/A1 reaction with either ING2 (2A) or CR2 (2B) antibodies. The peptide cleavage product indicating BoNT/A1 is present in both cases at *m/z* 1197.7 and the internal standard is present at *m/z* 1204.7.

The inhibition studies show that the catalytic activity of BoNT/A1 toxin is inhibited to some degree in the presence of any antibody (Table 2, Figure 1). The level of inhibition varies from slight to almost complete depending on the particular antibody used. For example, following mass spectrometric analysis, it is clear that there is a CT cleavage product at 1197.7 *m/z* in the reaction containing either the ING2 or CR2 antibodies (Figure 2). Both reactions contain the same amount of ISTD at *m/z* 1204.7, so by comparison of the size of the 1204.7 peaks with the size of the 1197.7 peaks, we can determine that the amount of CT cleavage product is much larger with the CR2 (2B) reaction than with the ING2 (2A) reaction. The sum of the results for BoNT/A1 indicate that the polyclonal antibody and mAbs ING2, 2G11, and polyclonal antibodies show between 95 and 100% inhibition, as they yield reactions with the least CT cleavage product. Mabs ING1 and 2A9 show between 75 and 85% inhibition of BoNT/A1 compared with other antibodies.

These differences can be partially explained through knowledge of the epitopes that these mAbs bind. The CR2 antibody binds an epitope on BoNT/A HC, far from the catalytic LC (Table 1) [27]. By contrast, the ING2 antibody binds an epitope on the LC (Table 1) [27] and inhibits the LC catalytic activity. Since the ING2 mAb is bound to the catalytically active portion of the toxin, the toxin is not as free to access its peptide substrate for cleavage. Note that not all of the LC binding mAbs are strongly inhibitory (e.g. 5A20.4 as evidenced in Table 2), indicating that the precise LC epitope bound determines whether there is catalytic inhibition or not.

ING1 and 2G11 are clonally related mAbs that bind identical epitopes requiring the presence of both the LC and the H_N_ toxin domains [27]. Since these mAbs do not have detectable binding to LC [27], we hypothesize that the mAbs hinder dissociation of the LC from the heavy chain, an event which many believe enhances catalysis. Both antibodies inhibit the activity of BoNT/A1, with the higher affinity antibody showing the greater inhibition (Table 2). The mechanism by which 2A9, a BoNT/A1 H_C_ binder, inhibits the reaction is not known. The polyclonal antibody is directed against many epitopes and it appears that some of these epitopes cause binding of the polyclonal antibody to regions of the BoNT/A1 molecule that also interfere with its ability to interact with its peptide substrate.

Figure 1 and Table 2 also depict the results of the reaction of these same antibodies with recombinant BoNT/A1 light chain alone. These data are consistent with that of BoNT/A1 complex, with a few exceptions: ING2, 2A9, and 5A20.4. All three of these antibodies have some inhibitory effect against BoNT/A1 complex, but do not appear to be very inhibitory against the light chain of BoNT/A1 alone.

The lack of LC inhibition by ING2 and 5A20.4 is difficult to explain, as both of these mAbs bind yeast displayed and recombinant BoNT/A LC [27, unpublished]. The translocation domain contains a belt-like region that wraps around the LC, holding it against the H_N_ in a way that prevents catalytic interaction with substrate [28]. One possible explanation is that in the intact neurotoxin these mAbs at least partially inhibit catalysis by preventing LC dissociation from the holotoxin, rather than by blocking substrate binding. Alternatively, the conformation of the recombinant light chain may be different from that in the BoNT/A1 complex in the reaction mixture such that ING2 and 5A20.4 cannot bind the recombinant light chain with as high affinity as the light chain within the BoNT/A1 complex. Monoclonal antibody 2A9 shows substantial inhibition of BoNT/A1, but not /A1 LC. This mAb binds to an undetermined epitope on the H_C_ portion of the toxin. The H_C_ portion is not present in the recombinant light chain, so this antibody cannot bind the toxin in order to inhibit it.

The BoNT/A2 protein differs from BoNT/A1 by 10% at the amino acid level. Because it is important to understand whether these genetic differences result in extraction or inhibition differences with various antibodies, the above panel of antibodies was also examined for inhibition of BoNT/A2. The data in Table 2 show that, despite genetic differences, most of the results for inhibition of BoNT/A2 are similar to that of BoNT/A1 complex, with the exception of the ING1 and CR2 antibodies. ING1 has a greater inhibitory effect against BoNT/A1 than it does against BoNT/A2. This may result from the 2.3-fold lower affinity for BoNT/A2.

By contrast, mAb 2G11, which was engineered for better cross-reactivity than ING1, has a much better affinity for both BoNT/A1 and /A2 (Table 1) and shows a high level of inhibition against both toxins. CR2, which was also engineered for better cross-reactivity, shows greater inhibition against BoNT/A2 than BoNT/A1. This contrasts with mAbs AR1-4, which show greater inhibition against BoNT/A1 than BoNT/A2 and were engineered for high affinity binding to BoNT/A1, not for better cross-reactivity to /A2 and /A3.

BoNT/A3 differs from BoNT/A1 by 15% at the amino acid level, and from BoNT/A2 by 6.3% [23], so we wanted to examine whether this panel of antibodies neutralized BoNT/A3 as well. The results are comparable to that of BoNT/A1 complex, with the exceptions of the ING1, ING2, and 2G11 antibodies (Table 2). Again, these antibodies are not as inhibitory toward BoNT/A3 as they are toward BoNT/A1 complex. ING2 binds BoNT/A1 and /A2 with low picomolar efficiency, but it does not bind /A3, and thus it does not inhibit the interaction of toxin with substrate. As mentioned above, 2G11 is a version of ING1 that was engineered for greater cross-reactivity among BoNT/A subtypes, and the success with this is seen in both the increased binding affinity (Table 1) and inhibition against both BoNT/A2 and /A3 compared with ING1.

### BoNT/A extraction efficiency

**Figure 3:**
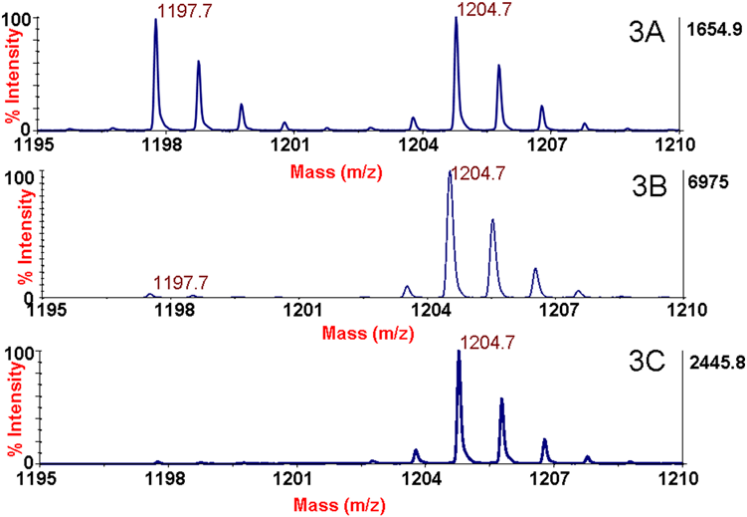
Mass spectra of the Endopep-MS botulinum neurotoxin A reaction after extraction of the toxin with either CR2 (3A), ING2 (3B), or B4 (3C) antibodies. The peptide cleavage product indicating the presence of BoNT/A is present in both cases at *m/z* 1197.7 and the internal standard is present at *m/z* 1204.7.

After determining which antibodies were more inhibitory toward the catalytic activity of BoNT/A1, /A2, and /A3, we wanted to examine the ability of the panel of antibodies in our assay to extract BoNT/A1, /A2, and /A3 in the Endopep-MS assay. All antibodies were used to extract the same level (100 mLD_50_) of BoNT/A from a buffer solution. Following extraction, the toxins on the beads were added to identical reaction mixtures containing peptide substrate. Upon mass spectrometric analysis, it is apparent (see Figure 3) that the CR2 (3A), ING2 (3B), and B4 (3C) antibody-extracted samples contain the internal standard at *m/z* 1204.7, but that only the CR2 and ING2 antibody-extracted samples contain C-terminal cleavage product at *m/z* 1197.7. Comparing the CT products with ISTDs shows that the CR2 antibody-extracted sample contains more C-terminal cleavage product than the B4 or ING2 antibody-extracted samples. Since all samples contain the same amount of internal standard, the generation of a higher level of cleavage product indicates a greater level of toxin, a greater activity of toxin, or possibly both.

Table 3 contains the peak area ratios of the cleavage product over the ISTD for 16 BoNT/A mAbs and one polyclonal antibody product used to extract either BoNT/A1, /A2, or /A3. mAbs ING1, ING2, 2A9, 2G11 and B4 yielded minimal cleavage product. Because the inhibitory experiments showed that the ING1, ING2, 2A9, and 2G11 antibodies inhibited the activity of BoNT/A1 complex, the decreased cleavage products after extraction with these antibodies are likely due to inhibition of activity rather than poor extraction efficiency. However, the inhibitory experiments determined that the decrease in BoNT/A1 activity in the presence of mAb B4 was equivalent to all other noninhibitory antibodies with the exception of CR2. Therefore, the decrease in cleavage product must be due to a decrease in the amount of toxin that was extracted rather than a decrease in the toxin's activity due to inhibition by the antibody. This poor extraction efficiency cannot be attributed to the binding affinity of B4 for BoNT/A1 (95.9 pM) but rather may reflect either poor coupling of the mAb to the beads or inactivation of the mAb upon coupling.

**Table 3:** Peak area ratios of the peptide cleavage product over the internal standard peptide obtained from the Endopep-MS reaction of BoNT A after its extraction by the antibody panel.

Antibody	BoNT A1	BoNT A2	BoNT A3
Polyclonal	0.31	0.15	0.24
2G11	0.02	0.06	0
ING2	0.02	0.09	0.06
ING1	0.12	0.21	0.12
2A9	0.02	0.03	0
AR2	1.03	0.27	0.12
CR1	1.1	0.75	1.02
RAZ1	1.07	1.17	0.66
AR4	1.31	0.39	0.42
5A20.4	0.32	0.03	0
AR1	0.88	0.15	0.12
B4	0	0	0
HuC25	1.09	0.33	0.36
4A1.1	1.03	0.03	0.3
C25	1.52	0.33	0.42
3D12	0.99	1.11	1.02
CR2	1.07	0.96	1.08

Three different forms of BoNT A were used, and they include A1, A2, and A3.

As with the inhibition studies, we also wanted to look at possible extraction efficiency differences due to genetic differences among the three BoNT/A subtypes. Only a few antibodies that had good extraction efficiency for BoNT/A1 also had good extraction efficiency for BoNT/A2 and BoNT/A3. These include CR1, CR2, 3D12, and RAZ1. Not surprisingly, the ability to extract the BoNT/A2 and BoNT/A3 subtypes was correlated with affinity. mAbs that did not bind these subtypes (4A1.1 and 5A20.4) exhibited no extraction. mAbs that bound BoNT/A2 and BoNT/A3 poorly (K_D_>200 nM, C25, AR1, AR2, and AR4) showed significantly less extraction than clonally related mAbs binding the same epitope with affinities less than 100 pM (CR1 and CR2). However the correlation of extraction efficiency with binding constant was not linear. At K_D_<100 pM, there were not significant differences in the extraction efficiencies of clonally related mAbs binding the same epitope (compare C25 vs AR4 for BoNT/A1 and 3D12 and RAZ1 for BoNT/A1, BoNT/A2, or BoNT/A3). This absence of effect likely reflects the binding rate constant. All of these mAbs have fast association rate constants (k_on_) and the K_D_ differences are largely due to differences in the dissociation rate constants (k_off_) [19]. However, the mAbs with the fastest k_off_ would remain associated with toxin with a half life of greater than 2 hours, thus not allowing time for the toxin to escape the bead capture before incubation with substrate.

## Discussion

The Endopep-MS assay relies upon antibody extraction of BoNT/A from a clinical or food sample as a sample preparation step prior to analysis for the toxin, so using the correct antibodies is a critical component of the assay. Because the assay is activity-based such that the toxin is detected through the enzymatic cleavage of target proteins, it is critical to include in the extraction step antibodies that do not hinder the activity of the toxin. Therefore, it is important to perform experiments to determine the inhibition ability of an antibody against BoNT/A prior to choosing an antibody for extracting BoNT/A. For the assay to be optimal, we would also like to choose an antibody that demonstrates a strong extraction efficiency for BoNT/A. Finally, the antibodies must have good extraction efficiencies for all known BoNT/A, which currently includes the BoNT/A1, /A2, /A3, and /A4 subtypes.

We therefore tested a panel of antibodies against BoNT/A1, /A2, and /A3 and examined both their inhibitory ability as well as their extraction efficiency. Antibodies that gave good results with all three toxin types were CR1, CR2, 3D12, and RAZ1. CR1 and CR2 bind the same C25 epitope, and the remaining two antibodies, 3D12 and RAZ1 bind a different non-overlapping epitope. It is known that using multiple antibodies that bind non-overlapping epitopes increases the effective affinity for the toxin by as much as 200-fold over the affinity of the individual antibodies [17]. Using multiple antibodies having different epitopes not only increases overall binding affinity, which is important for toxin extraction, but it also offers a unique opportunity to design a mixture of antibodies that bind a variety of epitopes, including regions that are conserved across the BoNT/A subtypes. This is important because we need the antibodies to be as inclusive as possible. Although only four subtypes of BoNT/A are currently known, it is possible that in the future that other BoNT subtypes may be discovered. While a new BoNT subtype may have an amino acid mutation in one epitope that prevents its extraction from a sample matrix by an antibody, the probability of mutation in two different epitopes is more remote. Therefore, using multiple antibodies against multiple epitopes is preferred for this assay.

From these studies, it appears that antibodies from two distinct epitopes that bind BoNT/A1, /A2, and /A3 with high affinity but do not inhibit the activity of the toxin would be the optimal antibodies to use in these assays. BoNT/A H_C_ epitopes appear to interfere with toxin activity less than LC or H_N_ epitopes, so we focused on H_C_ antibodies. The H_C_ epitopes showing the best toxin extraction efficiencies were the C25 and 3D12 epitopes.

Examining the calculated dissociation rate (K_D_) for BoNT/A1 and /A2 with the clonally related antibodies from these epitopes shows that CR2 has a substantially lower (better) K_D_, or dissociation equilibrium constant, for BoNT/A2 than CR1, and equivalent low K_D_s for BoNT/A1 are seen for both antibodies. In addition, CR2 is known to bind BoNT/A3 with a high affinity of 150 pM. The RAZ1 antibody shows a 40-fold increase in affinity for both BoNT/A1 and /A2 compared to the parental 3D12, and this antibody shows the highest overall affinity against all three BoNT/A subtypes with K_D_s ranging from 1.48–4.65 pM. Because of these findings, we have opted to use a mixture of RAZ1 and CR2 as the antibodies to extract BoNT/A1, /A2, and /A3 from sample matrices prior to analysis with the Endopep-MS method.

Because BoNT/A4 is produced in low levels, it was not feasible to test the entire antibody panel against BoNT/A4. However, RAZ1/CR2, the chosen antibody mixture, was tested against BoNT/A4 and has been found to efficiently extract BoNT/A4 for detection by Endopep-MS [12]. Thus, we have been able to show the RAZ1/CR2 antibody combination is effective at extracting and concentrating all known subtypes of BoNT/A prior to analysis by Endopep-MS.

In addition to determining the best antibodies for sample preparation prior to Endopep-MS, this work has determined *in vitro* inhibition abilities of a panel of antibodies against BoNT/A1, /A2, and /A3. Many antibodies show similar results with BoNT/A1, /A2, and /A3, but in some cases they differ, indicating differing toxin extraction efficiencies due to differing binding affinities or inhibition of toxin activity. These findings indicate that antibody choice is crucial to the ability of these types of assays to sensitively detect a diverse range of BoNT/A toxin subtypes, which is a critical first step to providing proper treatment in a timely manner. In addition, these antibody characterizations have the potential to assist with mechanistic studies of BoNT/A, which is important for studying alternative treatments for botulism.

## References

[1] Centers for Disease Control and Prevention (1899–1996). *Botulism in the United States: Handbook for Epidemiologists, Clinicians and Laboratory Workers*.

[2] Schiavo G, Matteoli M, Montecucco C (2000). Neurotoxins affecting neuroexocytosis.. Physiol Rev.

[3] Arnon SS, Schechter R, Inglesby TV, Henderson DA, Bartlett JG (2001). Botulinum toxin as a biological weapon: medical and public health management.. JAMA.

[4] Mahrhold S, Rummel A, Bigalke H, Davletov B, Binz T (2006). The synaptic vesicle protein 2C mediates the uptake of botulinum neurotoxin A into phrenic nerves.. FEBS Letters.

[5] Dong M, Yeh F, Tepp WH, Dean C, Johnson EA (2006). SV2 is the protein receptor for botulinum neurotoxin A.. Science.

[6] Simpson LL (2004). Identification of the major steps in botulinum toxin action.. Ann Rev Pharmacol Toxicol.

[7] Barr JR, Moura H, Boyer AE, Woolfitt AR, Kalb SR (2005). Botulinum neurotoxin detection and differentiation by mass spectrometry.. Emerg Infect Dis.

[8] Boyer AE, Moura H, Woolfitt AR, Kalb SR, Pavlopoulos A (2005). From the mouse to the mass spectrometer: detection and differentiation of the endoproteinase activities of botulinum neurotoxins A–G by mass spectrometry.. Anal Chem.

[9] Kalb SR, Moura H, Boyer AE, McWilliams LG, Pirkle JL (2006). The Use of Endopep-MS for the detection of botulinum neurotoxins A, B, E, and F in serum and stool samples.. Anal Biochem.

[10] Kalb SR, Goodnough MC, Malizio CJ, Pirkle JL, Barr JR (2005). Detection of botulinum neurotoxin A in a spiked milk sample with subtype identification through toxin proteomics.. Anal Chem.

[11] Gaunt PS, Kalb SR, Barr JR (2007). Detection of Botulinum Type E Toxin in channel catfish with visceral toxicosis syndrome using catfish bioassay and endopep mass spectrometry.. J Vet Diagn Invest.

[12] Kalb SR, Smith TJ, Moura H, Hill K, Lou J (2008). The Use of Endopep-MS to detect multiple subtypes of botulinum neurotoxins A, B, E, and F.. International Journal of Mass Spectrometry.

[13] Kautter DA, Solomon HM (1977). Collaborative study of a method for the detection of *Clostridium botulinum* and its toxins in foods.. J Assoc Anal Chem.

[14] Cenci Di Bello I, Poulain B, Shone CC, Tauc L, Dolly JO (1994). Antagonism of the intracellular action of botulinum neurotoxin type A with monoclonal antibodies that map to light-chain epitopes.. Eur J Biochem.

[15] Garcia-Rodriguez C, Levy R, Arndt JW, Forsyth CM, Razai A (2007). Molecular evolution of antibody cross-reactivity for two subtypes of type A botulinum neurotoxin.. Nat Biotechnol.

[16] Mah DC, Hu WG, Pon JK, Masri SA, Fulton RE (2003). Recombinant anti-botulinum neurotoxin A single-chain variable fragment antibody generated using a phage display system.. Hybrid Hybridomics.

[17] Nowakowski A, Wang C, Powers DB, Amersdorfer P, Smith TJ (2002). Potent neutralization of botulinum neurotoxin by recombinant oligoclonal antibody.. Proc Natl Acad Sci USA.

[18] Pless DD, Torres ER, Reinke EK, Bavari S (2001). High-affinity, protective antibodies to the binding domain of botulinum neurotoxin type A.. Infect Immun.

[19] Razai A, Garcia-Rodriguez C, Lou J, Geren IN, Forsyth CM (2005). Molecular evolution of antibody affinity for sensitive detection of botulinum neurotoxin type A.. J Mol Biol.

[20] Shone C, Wilton-Smith P, Appleton N, Hambleton P, Modi N (1985). Monoclonal antibody-based immunoassay for type A *Clostridium botulinum* toxin is comparable to the mouse bioassay.. Appl Environ Microbiol.

[21] Amersdorfer P, Wong C, Chen S, Smith T, Deshpande S (1997). Molecular characterization of murine humoral immune response to botulinum neurotoxin type A binding domain as assessed by using phage antibody libraries.. Infection Immunity.

[22] Amersdorfer P, Wong C, Smith T, Chen S, Desphande S (2002). Genetic and immunological comparison of anti-botulinum type A antibodies from immune and non-immune human phage libraries.. Vaccine.

[23] Hill KK, Smith TJ, Helma CH, Ticknor LO, Foley BT (2007). Genetic diversity among Botulinum Neurotoxin-producing clostridial strains.. J Bacteriol.

[24] Smith TJ, Lou J, Geren IN, Forsyth CM, Tsai R (2005). Sequence variation within botulinum neurotoxin serotypes impacts antibody binding and neutralization.. Infect Immun.

[25] Jensen MJ, Smith TJ, Ahmed SA (2003). Expression, purification, and efficacy of the type A botulinum neurotoxin catalytic domain fused to two translocation domain variants.. Toxicon.

[26] Geren IN, Lou J, Garcia C, Conrad F, Fan F Human monoclonal antibodies to botulinum neurotoxin types A and B from immune yeast displayed antibody libraries..

[27] Levy R, Forsyth CM, LaPorte SL, Geren IN, Smith LA (2007). Fine and domain-level epitope mapping of botulinum neurotoxin type a neutralizing antibodies by yeast surface display.. J Mol Biol.

[28] Lacy DB, Tepp W, Cohen AC, dasGupta BR, Stevens RC (1998). Crystal structure of botulinum neurotoxin type A and implications for toxicity.. Nat Struct Biol.

